# Academic outcomes and accommodations following adolescent sport-related concussion: a pilot study

**DOI:** 10.2217/cnc-2017-0009

**Published:** 2017-10-23

**Authors:** Kelly Russell, Erin Selci, Stephanie Chu, Adrian Rozbacher, Michael Ellis

**Affiliations:** 1 Department of Pediatrics & Child Health, University of Manitoba, Winnipeg, Canada; 2 Children's Health Research Institute of Manitoba, Winnipeg, Canada; 3 Canada North Concussion Network, Winnipeg, Canada; 4 College of Medicine, University of Manitoba; 5 Department of Surgery, University of Manitoba, Winnipeg, Canada

**Keywords:** academic outcomes, concussion, adolescents, school

## Abstract

**Aim:**

The purpose of this study was to examine academic achievement, absenteeism and school accommodations following adolescent sport-related concussion (SRC).

**Methods:**

A case-series was conducted among grade 8–12 students who suffered an SRC. The primary outcomes were change in pre- and post-concussion overall, core report card grade point average (GPA) and absenteeism due to concussion. The most helpful school accommodations were tabulated.

**Results:**

Pre- and post-concussion GPA was obtained from 33 students – 16 (48%) developed persistent post-concussion symptoms (symptoms lasting >4 weeks). There was no significant difference in pre- and post-concussion grades among students with a SRC for overall (p = 0.75) or core (p = 0.56) GPA. The median number of missed school days was 4 (interquartile range [IQR]: 2–8). Allowing for physical and cognitive rest was identified as the most helpful accommodation (30%).

**Conclusion:**

Larger studies should investigate the role that school accommodations and development of persistent post-concussion symptoms have on academic outcomes.

Concussion is an injury defined as “*a complex pathophysiological process affecting the brain, induced by biomechanical forces*” that typically results in clinical features that are attributable to alterations in brain functioning [1]. In 2009–2010, the incidence of head injury in Canadian youth ages 12–19 years was 554 per 100,000 with a significant proportion occurring during sports and recreational activities [2].

The clinical presentation of concussion is highly individualized and often includes a combination of physical, cognitive, sleep and emotional symptoms that have the potential to negatively impact a student's ability to attend and participate in school. While the majority of pediatric concussion patients will recover in 1–4 weeks, studies conducted across diverse clinical settings suggest that 30–74% will experience persistent postconcussive symptoms (PPCS) lasting longer than 1 month [3–8]. Patients who develop PPCS are at risk of experiencing impairments in physical, cognitive, emotional and social functioning and health-related quality of life [9–11].

Although a brief period of physical rest followed by a step-wise increase in physical activity is often successful in promoting a safe and timely return to sports following concussion, far less attention has focused on factors that can help guide the process of optimizing academic performance and school functioning following this injury. The school setting is often not conducive to cognitive rest and concussion recovery due to ongoing cognitive demands and exposure to certain environments (e.g., hallways and cafeterias) that may exacerbate concussion symptoms in certain patients [12]. Cognitive exertion can be reduced by individually tailored modifications to school attendance and workload, as well as by other accommodations such as permitting students to take breaks during classes [13]. Concussion can result in missed time from school and a recent systematic review found that those who developed post-concussion syndrome missed more school during recovery compared with those with an orthopedic injury [14]. Recognizing the challenges of returning to the classroom after concussion, the 5th International Conference on Concussion in Sport recently outlined a return-to-school strategy that outlines a four-step incremental process that assists student-athletes to make a gradual return to full school activities while monitoring their symptoms [15]. Although this is an important advance in concussion management, there has been little research to date that has focused on academic performance and school functioning following pediatric SRC.

Accordingly, the primary objective of this pilot study was to examine the effect of adolescent sport-related concussion (SRC) on pre- and post-concussion overall and core grade point average (GPA) as well as on the number of days missed from school; and to determine which academic school accommodations adolescent SRC patients received that were the most helpful in assisting with a successful return to school following this injury.

## Methods

### Study design & setting

We conducted a case-series pilot study among adolescents who presented to the Pan Am Concussion Program in Winnipeg, Manitoba, Canada. This pilot study was conducted to help inform the design of a future study examining academic and health-related quality of life outcomes in adolescents with SRC compared to orthopedic injuries. The Pan Am Concussion Program is a provincial government-funded, multidisciplinary pediatric concussion program that receives referrals from outside emergency departments as well as sports medicine and primary care physicians. At this facility, a single neurosurgeon is responsible for the evaluation and management of all pediatric concussion patients.

### Inclusion criteria & definitions

Adolescents were included in this study if they: sustained a physician-diagnosed SRC, were enrolled in a Winnipeg public or private school (grades 8–12), were concussed during the school year, and sustained their concussion within 30 days of their initial visit and were still symptomatic at the time of their initial visit. Both organized sports and recreational sports were included.

The diagnosis of SCR was made by the treating neurosurgeon and based on the definition set forth by the 4th international consensus statement on concussion in sport as an injury caused by biomechanical forces transmitted to the head that results in alterations in one or more clinical domains that include somatic symptoms, physical signs, behavioral changes, cognitive impairment and sleep disturbance [16]. At the time of the study, our pediatric concussion program did not have access to a clinical neuropsychologist. As such, neuropsychological testing was not used to supplement the diagnosis of SRC in these patients. Recent systematic reviews of outcomes following pediatric concussion suggested that the vast majority of pediatric concussion patients recovered within 1 month postinjury [8,17]. For the purpose of this study, PPCS was defined as persistent symptoms lasting greater than 4 weeks postinjury compared with baseline.

### Patient recruitment & data collection

Immediately prior to initial physician evaluation, the adolescent completed a standardized patient intake form (demographic information, patient history and a description of the concussion event). They also completed the post-concussion symptom scale (PCSS), a 22 item symptom inventory where patients rank symptom severity from 0 to 6. At the end of their initial evaluation, the treating neurosurgeon informed the adolescent and their parent(s) of their eligibility during the initial clinical visit. If the adolescent and parent(s) expressed interest, they then met with the Research Assistant (RA). The RA further explained the study and obtained informed parental consent and assent from the adolescent. Adolescents were then given a log book to track full or partial school days missed due to concussion symptoms, and were asked to provide a copy of their preconcussion report card (i.e., the report issued immediately prior to their concussion) that displays the teacher assigned grades.

Follow-up appointments were arranged weekly or as clinically indicated and not based on a prespecified protocol. Prior to their follow-up appointments, patients completed the PCSS, returned their completed attendance log book and were given a new log book. In general, patients were medically cleared to return to sport when they were asymptomatic or had returned to their preinjury baseline at rest, were tolerating full-time school, had a normal neurological examination [18] and completed the consensus guidelines return-to-play protocol [16].

Upon medically clearance, the RA asked youth to complete a 5 minute in-person exit survey that asked about school-related accommodations they received and if they found the accommodation beneficial as rated as most helpful, somewhat helpful, not helpful, or not available. Students were not asked to rank the accommodations. The list of 14 accommodations included attendance restrictions, workload reduction, extra time for exams and assignment, breaks from class, rescheduled tests, work in quiet environments, avoid taking notes, avoid computer work, avoid smartboards, avoid noisy environments, excused from or modified gym class, communication with teacher or nurses or other. At present there are no standardized, valid or reliable tools that have been developed to evaluate the perceived quality and adequacy of concussion-specific school accommodations. As such patients were asked to rate overall whether they felt the school academic accommodations they received were adequate or poor. The RA telephoned parents to request a copy of the post-concussion report card for the semester immediately after their child was deemed recovered. The following study took place at a time when there was no formalized institutional return-to-learn program or school accommodations provided to patients and parents.

### Analysis

For baseline characteristics, proportions were calculated for dichotomous data and means with standard deviations (SD) for normally distributed continuous data or medians with interquartile ranges for skewed continuous data. A t-test (or rank-sum test) was used to compare continuous characteristics of those who completed the study versus those who were lost to follow up, and a χ^2^ test was used for dichotomous data.

The primary outcomes of this study were change in mean pre- and post-concussion overall (all enrolled courses) and core (math, science, social studies, English and foreign languages) teacher assigned report grades and school absenteeism. Mean overall and core GPA were calculated with SD. A t-test was used to calculate the mean differences (with 95% CI and p-values) in students’ preconcussion versus post-concussion grades for their overall grades and the core subjects were calculated. In order to explore the effect of factors that could influence academic performance including sex, age, previous concussion history, symptom severity, school accommodations and school type, the results were stratified by sex, age (13–14 years vs 15–17 years), initial concussion symptom score quartiles (initial PCSS: 0–12, 13–33, 33+) previous concussion (yes/no), overall perception of school academic accommodations (adequate vs poor or somewhat adequate), public versus private school and subsequent development of PPCS (yes/no) among concussed adolescents. A p-value of less than 0.05 was interpreted as statistically significant. Insufficient sample size precluded performing multivariate linear regression analysis.

Absenteeism was tabulated from the attendance log book as the number of full days missed and the number of half days missed and expressed as median with interquartile range. The secondary outcome of this study was the assessment of school academic accommodations. The results of the exit survey inquiring about the type and quality of school-related accommodations were tabulated and proportions were calculated for each accommodation. The specific accommodation was interpreted as helpful if the student indicated it was ‘most helpful.’ Because this was a pilot study of the proposed methodology for evaluating academic performance and accommodations in this clinical population a sample size power calculation was not carried out.

### Ethical approval

Ethical approval was granted by the University of Manitoba.

## Results

### Baseline characteristics

In total, 48 adolescent athletes were recruited; however, three were lost to follow up and 12 submitted only one report card. Complete pre- and post-concussion GPA was obtained from 33 participants: 19 (57.6%) were male, 19 (57.6%) sustained a previous concussion and 15 (45.4%) subsequently developed PPCS. All patients were seen within 24 days of their injury and the median days to appointment were 8 (IQR: 5, 13). Their initial median PCSS was 27 (IQR: 8, 35). All students had at least 1 month between concussion and their post-concussion report card being issued (median: 72 days; IQR: 55, 139). Additional baseline characteristics appear in Table 1. With the exception of age, there was no significant differences in baseline characteristics among those with complete report card data and those who were lost to follow-up or only submitted one report card. Adolescents who were lost to follow up or had incomplete data were significantly older than those with complete data (0.64 years; 95% CI: 0.06, 1.23).

**Table 1.** **Baseline characteristics of concussed students (n = 33).**

**Characteristic**	**n (%)^†^**
Age (mean years, SD)	14.3 (1.0)

Male	19 (57.6)

History of ADHD	3 (9.1)

History of migraine or nonspecific headaches	3 (9.1)

Previous concussion	19 (57.6)

Initial PCSS (median IQR)	27 (8, 35)

Loss of consciousness	7 (22.6)

***Sport played at time of concussion***	

– Hockey	9 (27.3)

– Soccer	5 (15.2)

– Football	4 (12.1)

– Snowboard	3 (9.1)

– Other sport	12 (36.4)

Diagnosed with PPCS	15 (45.5)

Visits until recovered (median IQR)	4 (3, 5)

Days to recovery (median IQR)	35 (21, 56)

^†^Unless otherwise specified.

ADHD: Attention deficit hyperactivity disorder; PCSS: post-concussion symptom scale; PPCS: Postconcussive symptom; SD: Standard deviation.

### Change in overall & core GPA

The overall GPA was 82.9% (SD 8.5%) preconcussion and 82.7% (SD 8.0%) after concussion recovery (mean change: -0.2%, 95% CI: -1.6%, 1.1%). The preconcussion core GPA was 80.0% (SD 10.1%) and decreased to 79.4% (SD 10.4%) after concussion recovery (difference: -0.6%, 95% CI: -2.8%, 1.6%). Stratification by sex, age, history of previous concussion, initial PCSS tertile, public or private school, subsequent development of PPCS or patient-reported quality of school academic accommodations showed no statistically significant changes in overall or core GPA (Table 2).

**Table 2.** **Change in overall and core grade point average with 95% confidence intervals.**

	**GPA**	**Mean GPA (95% CI)**		**Mean% difference (95% CI)**	**p-value**

		***Preconcussion***	***Post-concussion***		
All concussion (n = 33)	Overall	82.91 (79.92, 85.91)	82.70 (79.87, 85.53)	-0.21 (-1.57, 1.14)	0.75

	Core	80.03 (76.44, 83.62)	79.40 (75.72, 83.08)	-0.63 (-2.81, 1.56)	0.56

***Sex***					

Male (n = 19)	Overall	80.95 (77.12, 84.78)	80.51 (77.13, 83.88)	-0.44 (-2.26, 1.38)	0.62

	Core	77.99 (73.64, 82.33)	77.18 (72.23, 82.12)	-0.81 (-72, 2.10)	0.57

Female (n = 14)	Overall	85.58 (80.58, 90.58)	85.67 (80.76, 90.57)	0.09 (-2.25, 2.43)	0.93

	Core	82.80 (76.32, 89.27)	82.41 (76.57, 88.26)	-0.38 (-4.15, 3.39)	0.83

***Age***					

13–14 years (n = 20)	Overall	81.83 (78.37, 85.29)	81.84 (78.86, 84.81)	0.01 (-2.11, 2.12)	0.97

	Core	78.84 (74.53, 83.14)	79.13 (75.90, 82.36)	0.30 (-2.56, 3.16)	0.83

15–17 years (n = 13)	Overall	84.57 (78.56, 90.58)	84.02 (77.90, 90.14)	-0.55 (-2.12, 1.01)	0.46

	Core	81.87 (74.89, 88.84)	79.81 (71.02, 88.60)	-2.05 (-5.80, 1.69)	0.25

***Initial symptom severity***					

PCSS 0–12 (n = 12)	Overall	84.03 (78.28, 89.77)	85.33 (80.56, 90.09)	1.30 (-0.79, 3.40)	0.19

	Core	82.90 (77.26, 88.53)	83.47 (78.45, 88.51)	0.58 (-2.49, 3.65)	0.68

PCSS 13–32 (n = 7)	Overall	84.51 (78.01, 91.01)	83.58 (77.71, 89.45)	-0.93 (-4.11, 2.25)	0.53

	Core	80.69 (72.52, 88.86)	79.96 (73.82, 86.09)	-0.73 (-4.33, 2.87)	0.66

PCSS 33+ (n = 14)	Overall	79.81 (74.47, 85.15)	77.98 (72.91, 83.05)	-1.83 (-4.09, 0.44)	0.10

	Core	75.29 (68.23, 82.35)	73.9 (64.10, 82.08)	-2.20 (-8.43, 4.03)	0.44

***Previous concussion***					

No history (n = 14)	Overall	84.68 (80.08, 89.27)	84.79 (80.22, 89.36)	0.11 (-2.33, 2.55)	0.92

	Core	81.40 (75.97, 86.83)	82.25 (77.44, 87.06)	0.84 (-1.191, 3.61)	0.52

History (n = 19)	Overall	81.61 (77.37, 85.84)	81.16 (77.36, 84.95)	-0.45 (-2.21, 1.30)	0.59

	Core	79.02 (73.83, 84.21)	77.30 (71.80, 82.81)	-1.72 (-5.05, 1.62)	0.29

***Private school***					

Private (n = 8)	Overall	82.86 (75.90, 89.81)	83.06 (77.63, 88.48)	0.20 (-2.89, 3.29)	0.89

	Core	79.56 (70.53, 88.59)	80.51 (73.36, 87.65)	0.95 (-3.07, 4.96)	0.59

Public (n = 25)	Overall	82.93 (79.35, 86.51)	82.58 (79.07, 86.10)	-0.35 (-1.98, 1.29)	0.67

	Core	80.18 (76.00, 84.36)	79.04 (74.49, 83.60)	-1.1324 (-3.83, 1.57)	0.40

***School accommodation***					

Adequate (n = 20)	Overall	82.79 (78.86, 86.73)	82.92 (79.38, 86.45)	0.13 (-1.14, 1.39)	0.84

	Core	79.73 (75.03, 84.42)	80.43 (76.38, 84.48)	0.70 (-1.05, 2.45)	0.41

Poor (n = 13)	Overall	83.09 (77.75, 88.44)	82.36 (76.98, 87.74)	-0.73 (-3.89, 2.42)	0.62

	Core	80.50 (74.04, 86.96)	77.82 (70.08, 85.57)	-2.67 (-7.79, 2.44)	0.27

***PPCS (symptoms greater than 28 days)***					

PCS (n = 15)	Overall	81.92 (78.12, 85.72)	80.72 (76.83, 84.62)	-1.20 (-3.51, 1.12)	0.25

	Core	77.13 (71.68, 82.59)	75.70 (69.48, 81.92)	-1.43 (-5.44 2.58)	0.44

No PCS (n = 18)	Overall	83.74 (78.91, 88.56)	84.34 (80.01, 88.58)	0.60 (-1.10, 2.31)	0.41

	Core	82.44 (77.48, 87.40)	82.48 (78.13, 86.84)	0.04 (-2.68, 2.84)	0.95

### Absenteeism & academic accommodations

The median number of missed school days was 4 (IQR 2–8 days). Thirty of the 33 students completed exit survey indicating which school accommodations they found most useful. The most helpful accommodation indicated by these students included cognitive and physical rest (30.3%, n = 10), having less work or more time to do their work (24.2%, n = 8), and being allowed to slowly return to school (15.2%, n = 5). Other accommodations rated as most helpful included taking breaks in school (9.1%, n = 3), having a positive and supportive environment at school (6.1%, n = 2), having good communication between home and school (3.0%, n = 1) and using the computer/smartboard less (3.0%, n = 1). 13 participants (39%) rated the overall quality of their school accommodations as poor.

## Discussion

The present study provides important insight into the effects of adolescent SRC on school functioning and objective measures of academic performance.

First, when preconcussion overall or core GPA was compared with post-concussion GPA, there was no significant difference in teacher assigned report card grades among adolescent SRC patients. A recent systematic review suggests that very few studies to date have directly analyzed GPA following a concussion as a measure of objective academic performance and compared these results to preinjury records or to noninjured or other injury controls [19]. Among these available studies, three found no significant decrease in GPA after sustaining a concussion or no significant differences when comparing concussed students to noninjured or other injury controls [20–22]. One study from South Africa found a significant decrease in academic performance in the subject Afrikaans [23] but no difference across other school subjects. Our recent retrospective population-based study of Manitoba children and adolescents found no significant decrease in end of year teacher assigned grades after a concussion compared with school- and grade-matched controls without a concussion [24]. Taken together, the results from our systematic review, retrospective population-based study and prospective case-series indicate that adolescent concussion has minimal effect on academic performance as measured by change in school grades.

Second, in the present study we found that students who sustained a SRC missed less than 1 week of school. These results are consistent with previous research that found that similarly aged students missed between 2 and 5 days of school while recovering from a concussion [25–27]. However, one study conducted at a tertiary pediatric concussion clinic observed a median time to returning to school part-time of 12 days and median time to returning to school full-time without academic accommodations of 35 days [4]. These findings suggest that the effect of pediatric SRC on school functioning is highly variable and likely impacted by individual patient factors including injury severity and rate of recovery. Although the proportion of PPCS development in this study is within the range observed among populations who present to pediatric tertiary concussion clinics [4,28], it is slightly higher than rates observed in previous studies examining academic outcomes among patients evaluated in the emergency department setting [17,25]. Although students with PPCS did not perform significantly worse academically than those who did not subsequently develop PPCS, the clinical manifestations of PPCS (e.g., vestibulo–ocular dysfunction, migraine headaches, postinjury psychiatric outcomes) may present unique challenges that impact school functioning and quality of life over a longer period of time, and thus warrant further investigation [29,30].

Third, this study provided valuable insight into school academic accommodations that may benefit patients returning to the classroom following SRC. Students in our study reported the most helpful accommodations to be engaging in cognitive and physical rest and having more time to complete work or a reduced workload. These findings are in agreement with a previous questionnaire-based study that found that 87% of concussed students were excused from physical activity, 87% received extensions on assignments, 84% were allowed excused absences, 84% received rest periods, 75% were able to postpone tests, 74% received extended testing time, 73% were permitted a reduced workload and 64% received accommodation for light and noise [31]. Taken together, the results of this pilot study have helped contribute to the development of an individually tailored return to learn (RTL) program at our tertiary pediatric concussion program, however future work is needed to evaluate the benefit of this RTL program in optimizing academic performance and accommodations following pediatric concussion.

### Limitations

There are several limitations to this study and the results should be interpreted cautiously. First, the greatest limitation of this study is the small sample size and that nonstatistically significant results must be interpreted cautiously. Although the small sample size resulted in an underpowered study to detect a statistical difference between pre- and postinjury grades (*post hoc* power calculations ranging from 10 to 20%), the observed absolute differences in overall and core GPAs ranging from 0.01 to 2.20 are unlikely to have resulted in any real world impact on passing courses or grade graduation rates. The small sample size in this study was impacted primarily by difficulties in obtaining both pre- and post-concussion report cards. The RAs telephoned parents a maximum of six times to ask for their child's report card. This study limitation is common to other previous studies examining academic outcomes in concussion patients that noted similar challenges obtaining school records due to consent, compliance or gaps in testing [20,21]. A second limitation is the variability in timing of concussion and recovery within the school semesters. Students who have preconcussion and post-concussion grades fall before and after summer may benefit from the additional recovery time in an environment more conducive to cognitive rest than the classroom. Students whose preconcussion and post-concussion grades fall in different terms or semesters may have different courses and this prevented the calculation of course specific change in GPAs. Students who suffered a concussion in one semester may have also changed their next semester courses to include easier classes and this would result in inconsistencies in difficulty and workload. Also, it was not feasible to control for the same course enrollment, number of tests, assessments or projects prior to or after the concussion. Despite these possibilities, the core GPA consisted of more challenging courses and it did not significantly decrease after a concussion. A fourth limitation of the study is that all SRC patients were recruited from a pediatric multidisciplinary concussion program that may have selected for students with more severe injuries and that may have taken longer to recover following injury. As such, the findings of this study may be generalizable to patients presenting to tertiary concussion clinics but not to a more generalized population of adolescent concussion patients. Last, differences in grading systems between school divisions, public versus private schools and individual teachers as well as variability in assigned work may have also impacted the results; however, this variation should be randomly distributed across study participants.

### Conclusion & future perspective

While adolescent SRC patients did not experience any evidence of significant academic decline as measured by report card grades, this study highlights important challenges that must be considered for future studies that aim to identify clinical variables that place select students at risk for worse academic outcomes following concussion. Further research is needed to allow physicians, parents, teachers and school administrators to work together to better assist students with concussion to make a successful return to school. In some instances, individualized learning plans and return-to-learn programs may be needed to optimize recovery, minimize absenteeism and maintain preconcussion academic performance.

Further studies that compare grades before and after a concussion using a control group and a larger sample size are essential to further understanding the effect of concussion on school functioning and academic outcomes. Additional work is also need to evaluate the benefit of individually tailored return-to-learn programs and school accommodations on objective measures of school functioning and academic outcomes. Future studies must also consider the wide range of patient and school factors that can impact the academic performance and school functioning following concussion.

**Executive summary**

- Academic achievement, absenteeism and school accommodations following adolescent sport-related concussion have been understudied.
- In a case-series of 33 students in grades 8–12, there was no significant difference in pre and post-concussion teacher-assigned report card grades.
- There was no significant difference in core grades (English, math, sciences, social studies, or foreign language).
- Students missed a median of 4 days of school.
- Physical and cognitive rest were the most useful school accommodations.
